# Supplementary material for: Antibiotics promote intestinal growth of carbapenem-resistant Enterobacteriaceae by enriching nutrients and depleting microbial metabolites
Source: Nat Commun. 2023 Aug 22;14:5094. doi: 10.1038/s41467-023-40872-z (PMC10444851; doi:10.1038/s41467-023-40872-z)
Supplement: Supplementary file 1 — Supplementary Information [file 41467_2023_40872_MOESM1_ESM.pdf]

SUPPLEMENTARY INFORMATION

SUPPLEMENTARY FIGURES

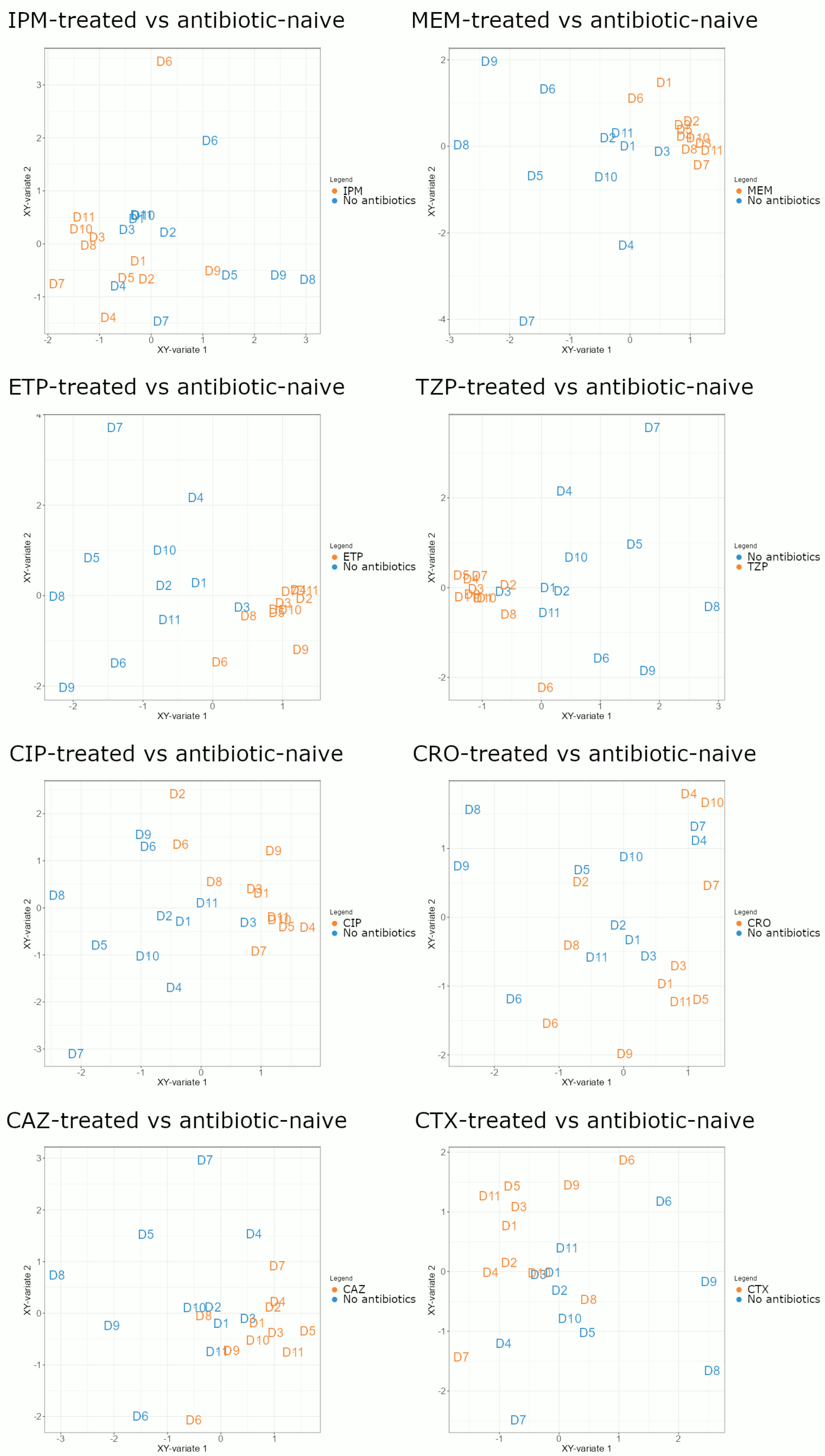

Figure S1: Antibiotic treatment resulted in a separation of antibiotic-treated and antibiotic-naïve human faecal microbiota in rCCA representation unit plots. rCCA models correlating 16S rRNA gene sequencing data (family level) and ^{1}H-NMR spectroscopy data. Representation of units (aka samples) for the first 2 canonical variates showing the correlations between variables antibiotic-treated (orange) and antibiotic-naïve (blue). n = 11 healthy faecal donors, D1-D11 = samples from donor number 1-11. Imipenem/cilastatin, IPM; meropenem, MEM; ertapenem, ETP; piperacillin/tazobactam, TZP; ciprofloxacin, CIP; ceftriaxone, CRO; ceftazidime, CAZ; cefotaxime, CTX. Source data are provided as a Source Data file.

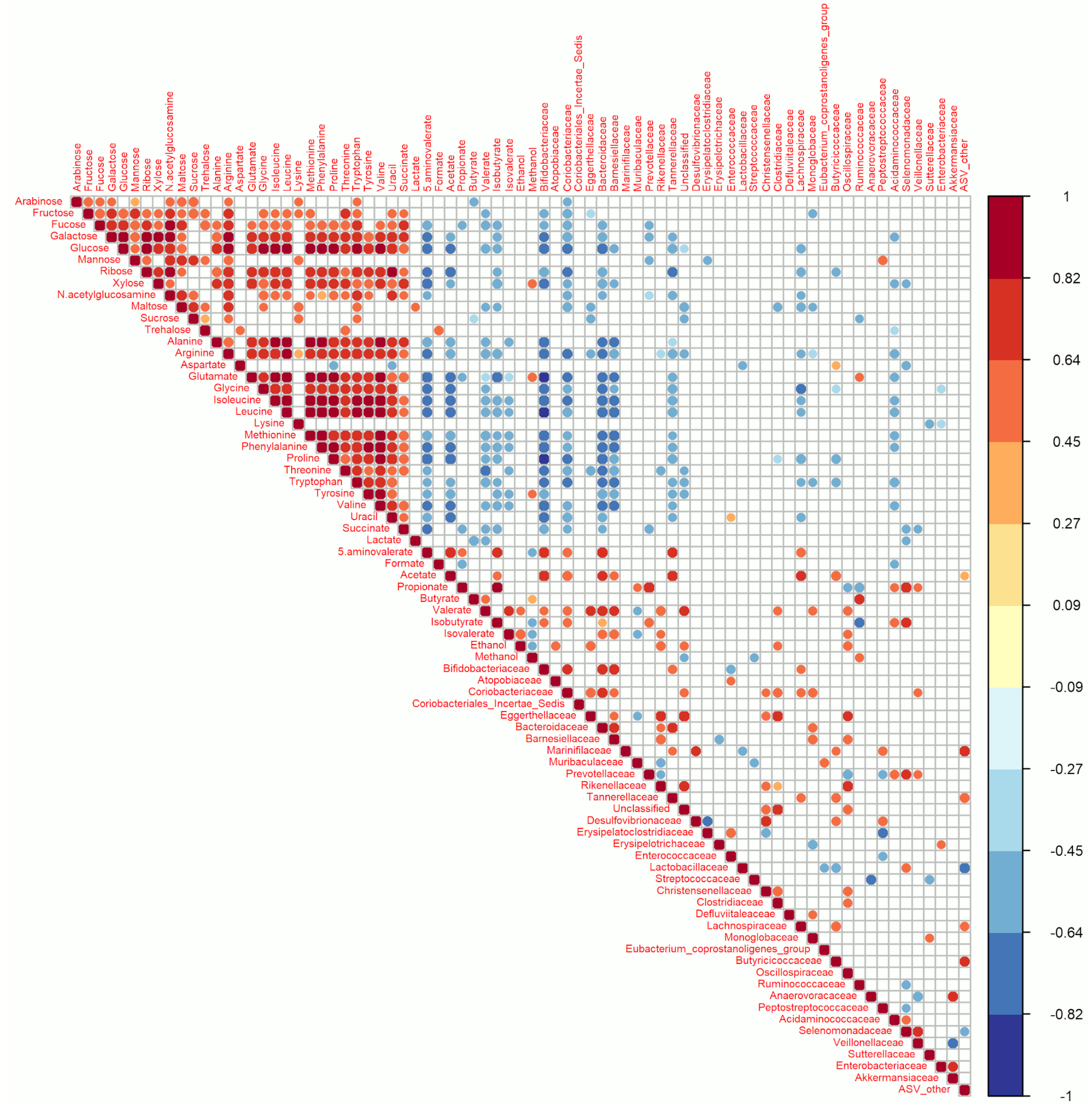

Figure S2: Correlation heatmap representing partial Spearman correlation coefficients between ^{1}H-NMR spectroscopy data and 16S rRNA gene sequencing data (family level) to investigate meropenem (MEM) treatment on faecal culture samples. Red represents positive correlations and blue represents negative correlations. Correlation coefficients with p > 0.05 are not plotted. Source data are provided as a Source Data file.

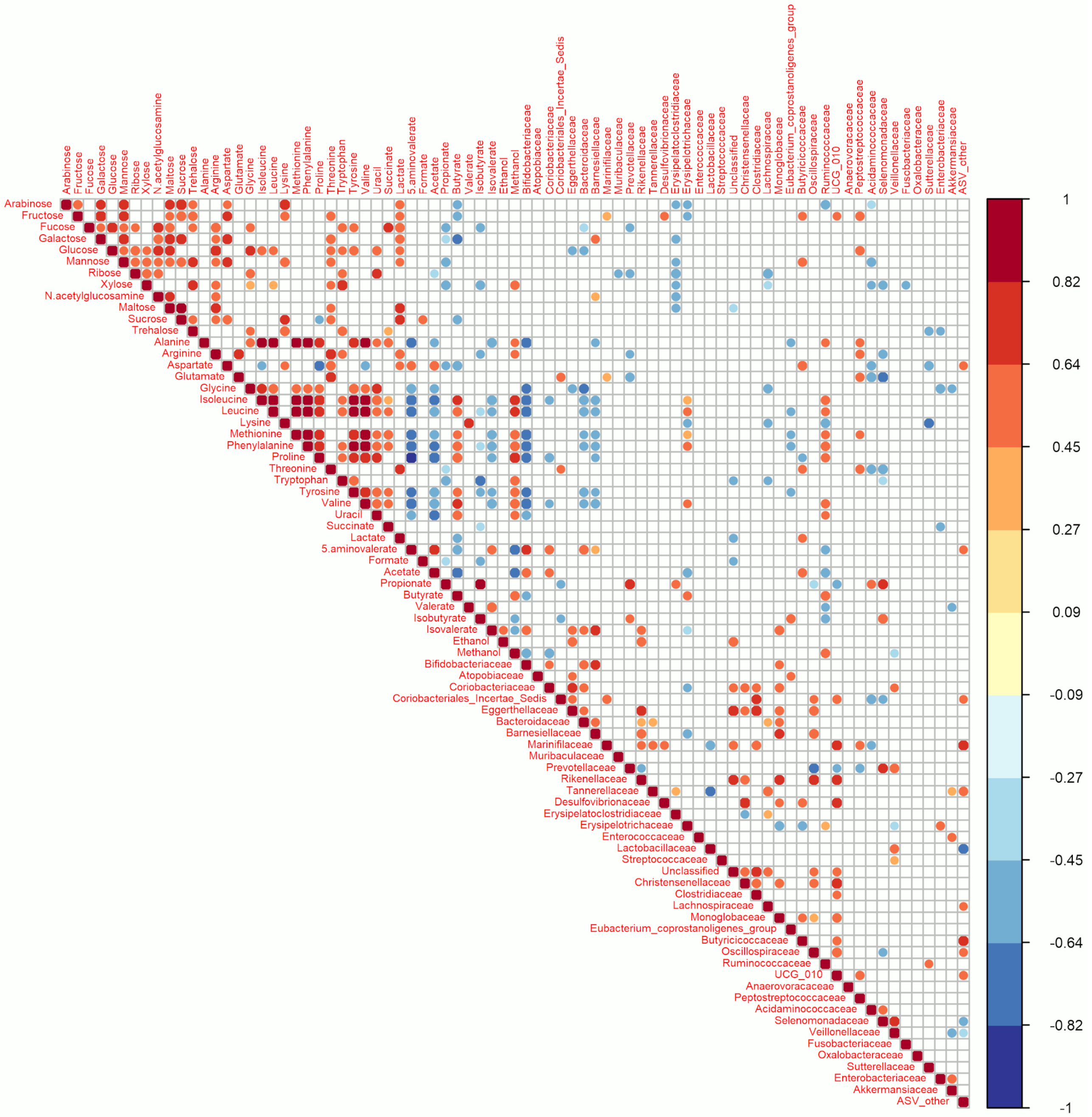

Figure S3: Correlation heatmap representing partial Spearman correlation coefficients between ^{1}H-NMR spectroscopy data and 16S rRNA gene sequencing data (family level) to investigate imipenem/cilastatin (IPM) treatment on faecal culture samples. Red represents positive correlations and blue represents negative correlations. Correlation coefficients with $p > 0.05$ are not plotted. Source data are provided as a Source Data file.

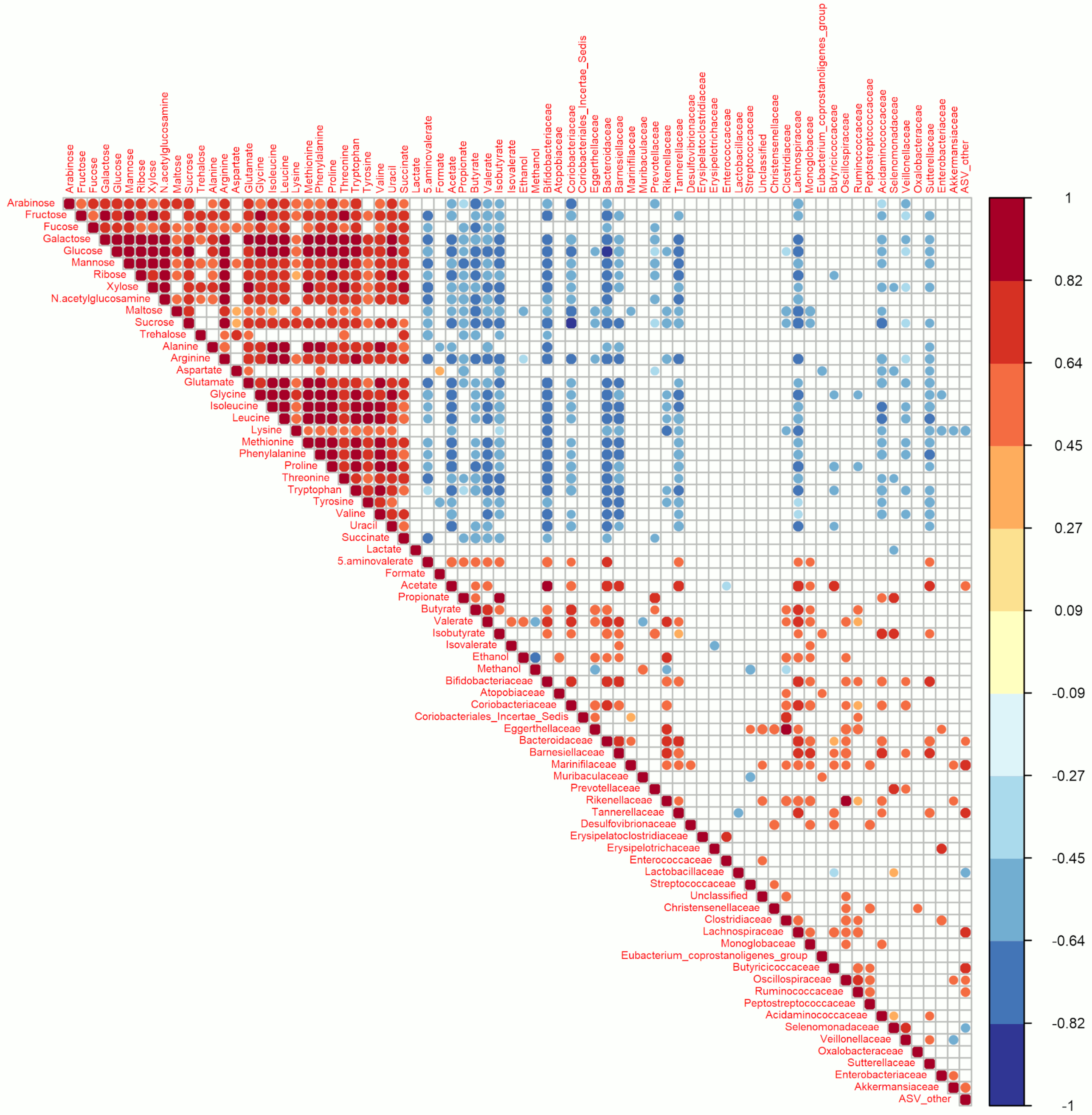

Figure S4: Correlation heatmap representing partial Spearman correlation coefficients between ^{1}H-NMR spectroscopy data and 16S rRNA gene sequencing data (family level) to investigate ertapenem (ETP) treatment on faecal culture samples. Red represents positive correlations and blue represents negative correlations. Correlation coefficients with p > 0.05 are not plotted. Source data are provided as a Source Data file.

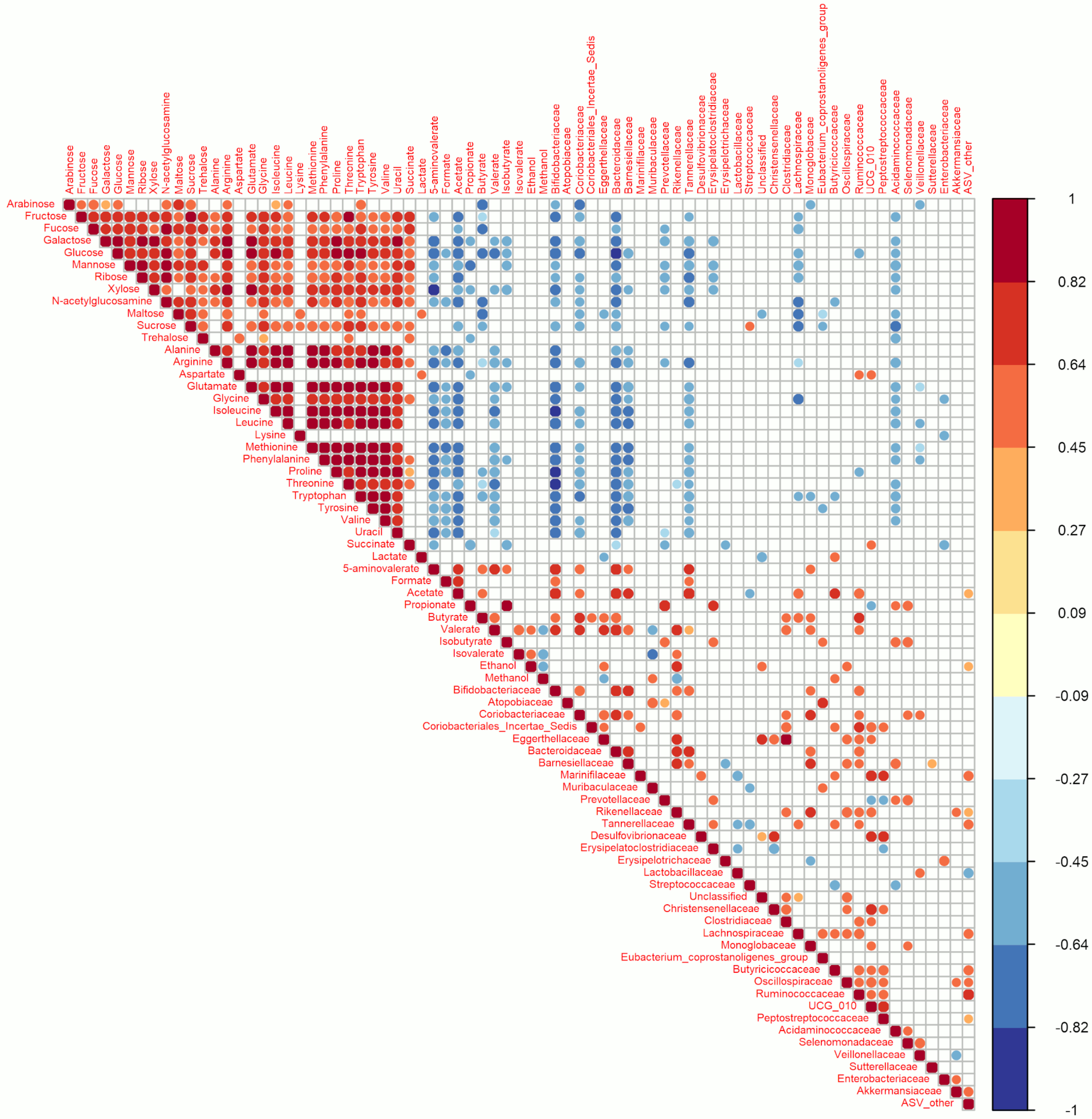

Figure S5: Correlation heatmap representing partial Spearman correlation coefficients between ^{1}H-NMR spectroscopy data and 16S rRNA gene sequencing data (family level) to investigate piperacillin/tazobactam (TZP) treatment on faecal culture samples. Red represents positive correlations and blue represents negative correlations. Correlation coefficients with $p > 0.05$ are not plotted. Source data are provided as a Source Data file.

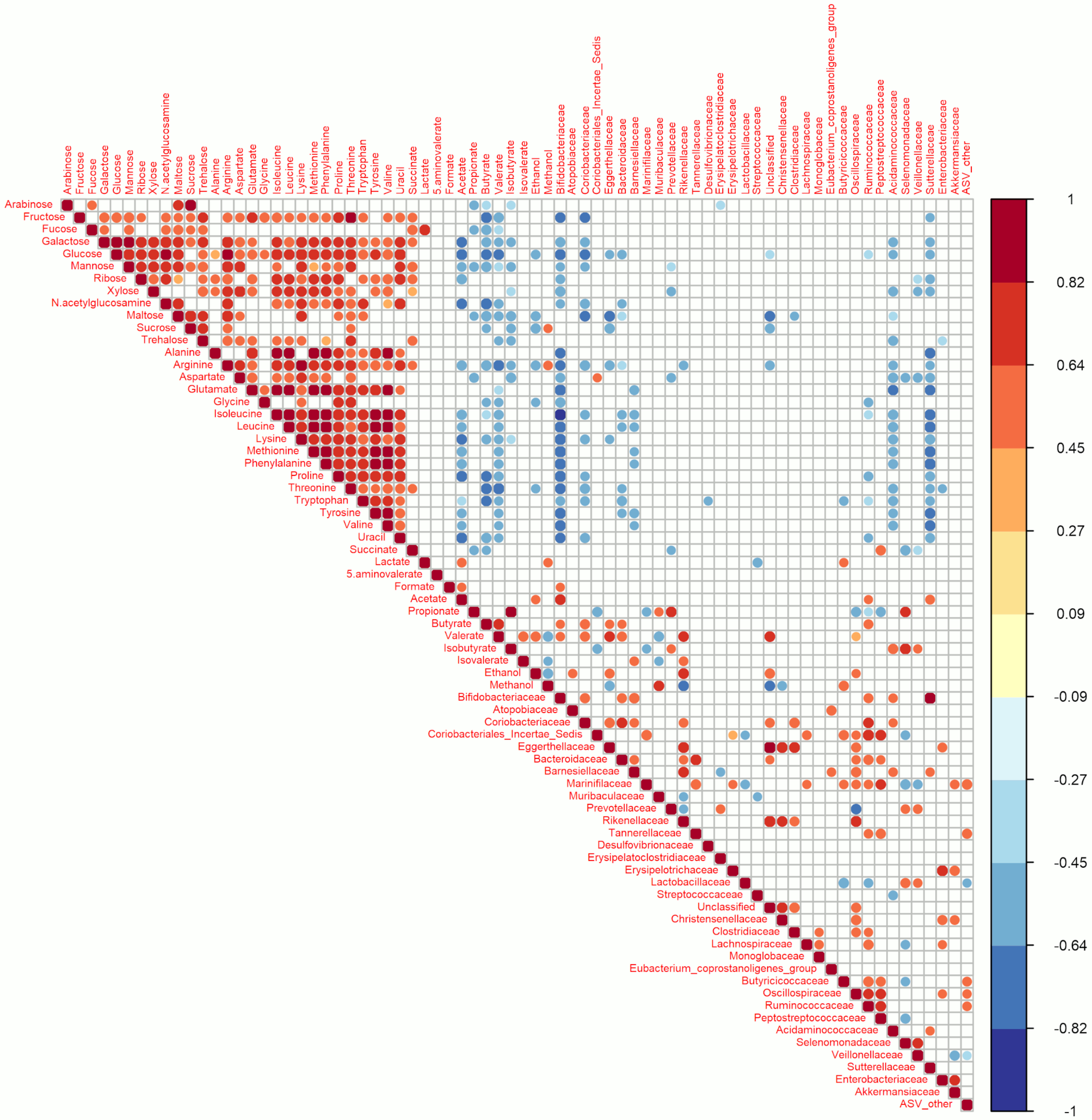

Figure S6: Correlation heatmap representing partial Spearman correlation coefficients between ^{1}H-NMR spectroscopy data and 16S rRNA gene sequencing data (family level) to investigate ciprofloxacin (CIP) treatment on faecal culture samples. Red represents positive correlations and blue represents negative correlations. Correlation coefficients with $p > 0.05$ are not plotted. Source data are provided as a Source Data file.

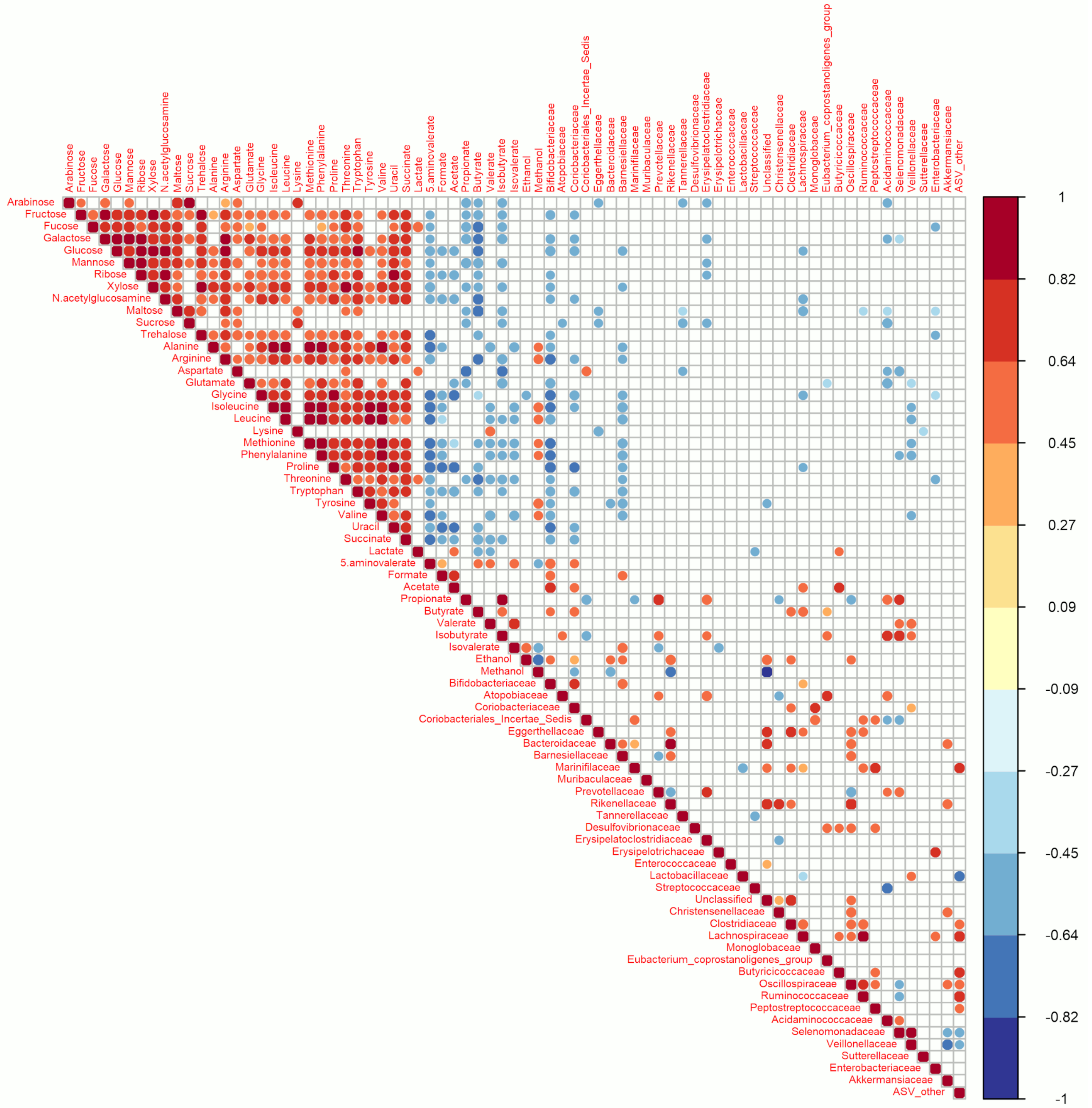

Figure S7: Correlation heatmap representing partial Spearman correlation coefficients between ^{1}H-NMR spectroscopy data and 16S rRNA gene sequencing data (family level) to investigate ceftriaxone (CRO) treatment on faecal culture samples. Red represents positive correlations and blue represents negative correlations. Correlation coefficients with $p > 0.05$ are not plotted. Source data are provided as a Source Data file.

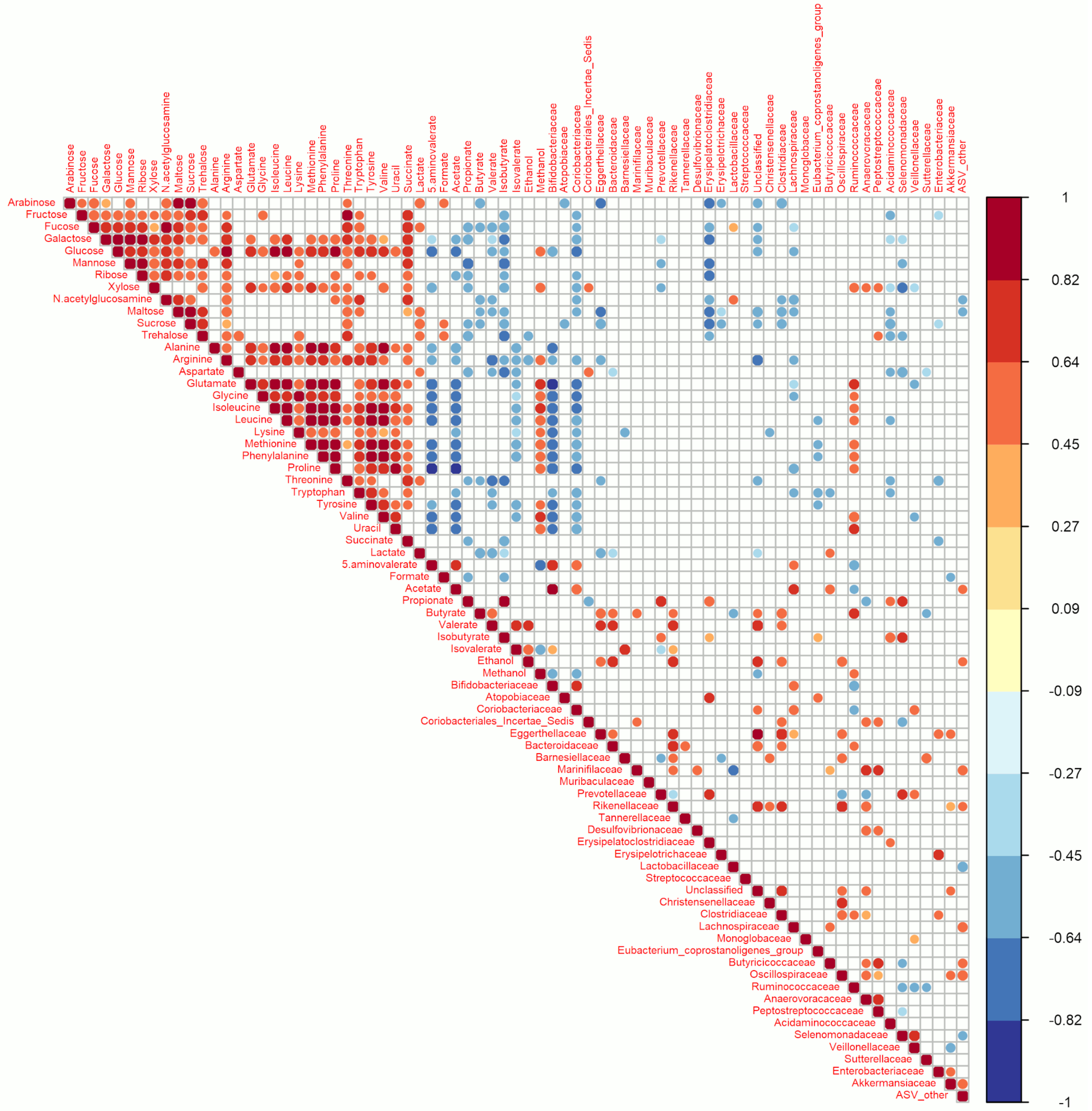

Figure S8: Correlation heatmap representing partial Spearman correlation coefficients between ^{1}H-NMR spectroscopy data and 16S rRNA gene sequencing data (family level) to investigate ceftazidime (CAZ) treatment on faecal culture samples. Red represents positive correlations and blue represents negative correlations. Correlation coefficients with $p > 0.05$ are not plotted. Source data are provided as a Source Data file.

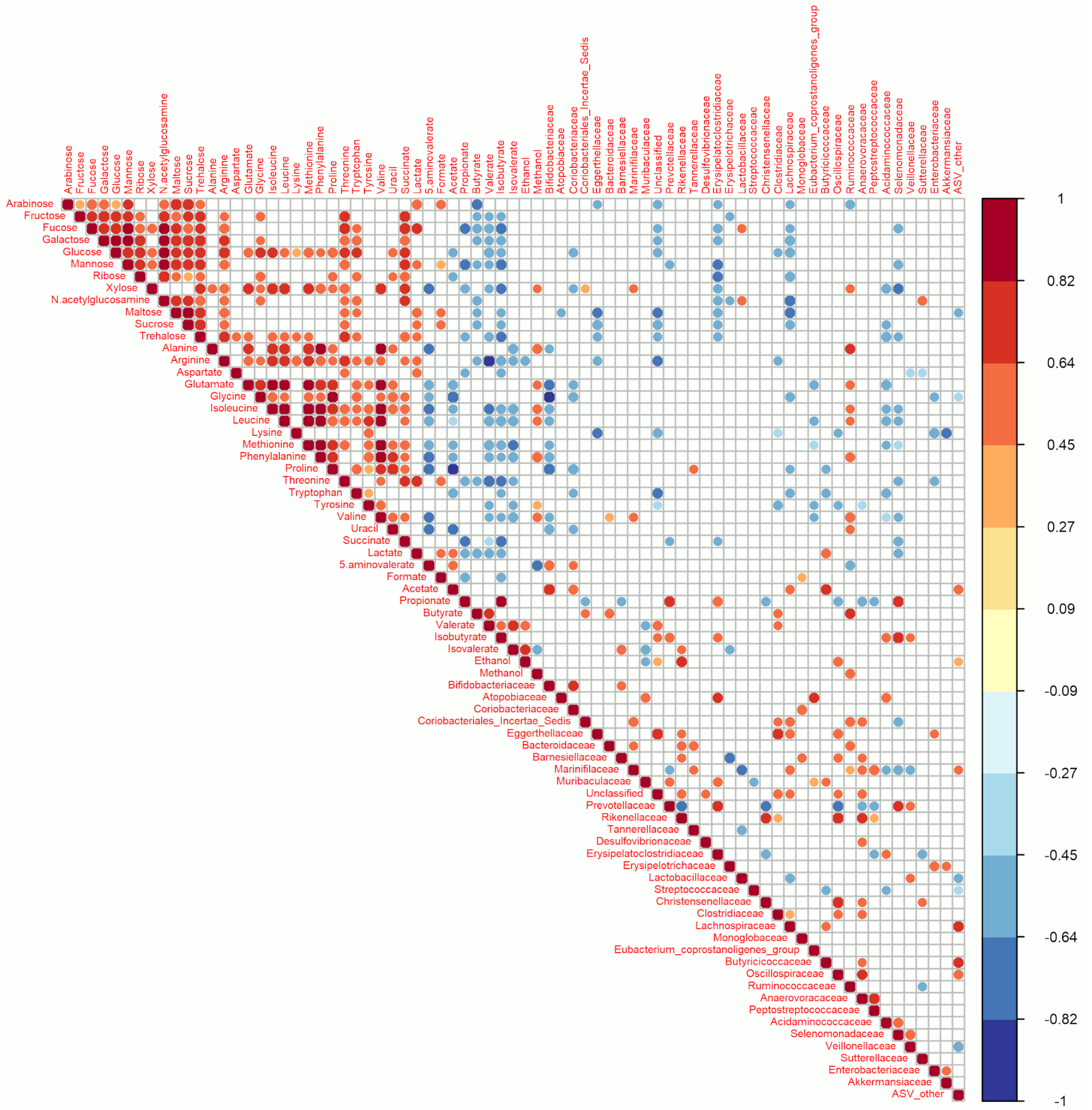

Figure S9: Correlation heatmap representing partial Spearman correlation coefficients between ^{1}H-NMR spectroscopy data and 16S rRNA gene sequencing data (family level) to investigate cefotaxime (CTX) treatment on faecal culture samples. Red represents positive correlations and blue represents negative correlations. Correlation coefficients with p > 0.05 are not plotted. Source data are provided as a Source Data file.

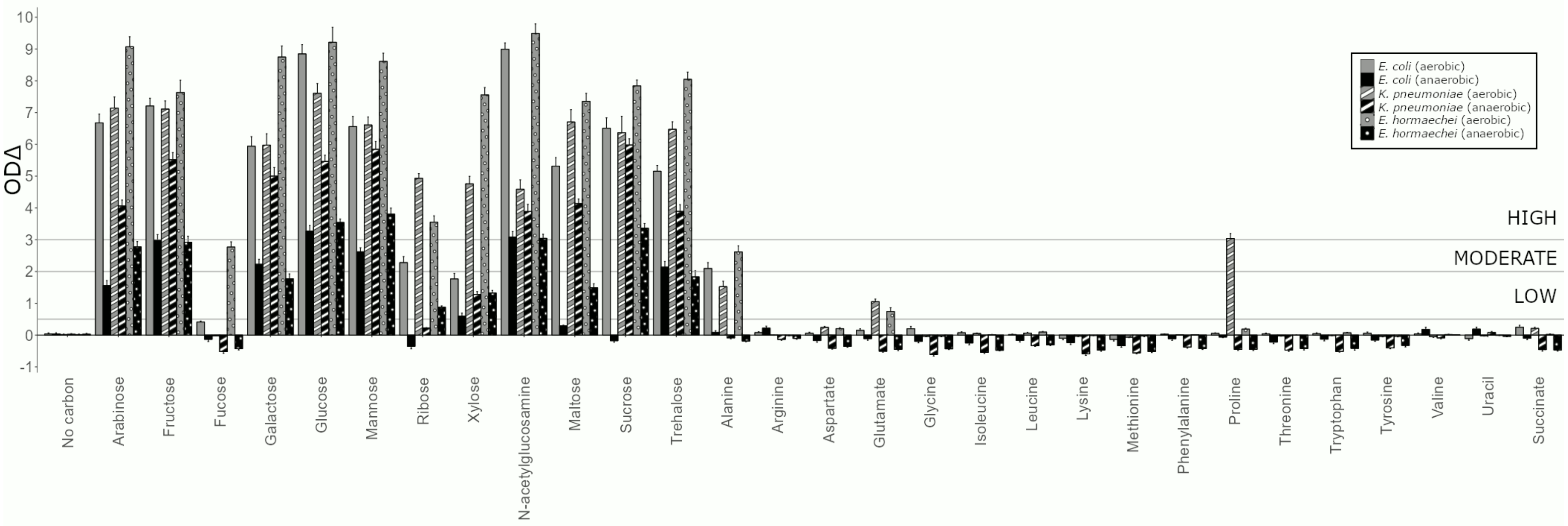

Figure S10: Individual carbon sources support the growth of CRE at high, moderate, or low levels. Functional differences (ODΔ) between growth on a carbon source versus growth on the no carbon control for *E. coli* ST617, *K. pneumoniae* ST1026, and *E. hormaechei* ST278 grown under anaerobic or aerobic conditions are summarised with the sum of functional differences quantifying the magnitude of differences between two growth curves. ODΔ >3 indicated high growth, ODΔ between 2-3 indicated moderate growth, ODΔ between 0.5-2 indicated low growth, ODΔ <0.5 indicated negligible or no growth. Error bars indicate 95% confidence intervals. Growth of each isolate was measured with 6 replicates in 2-3 independent experiments. Source data are provided as a Source Data file.

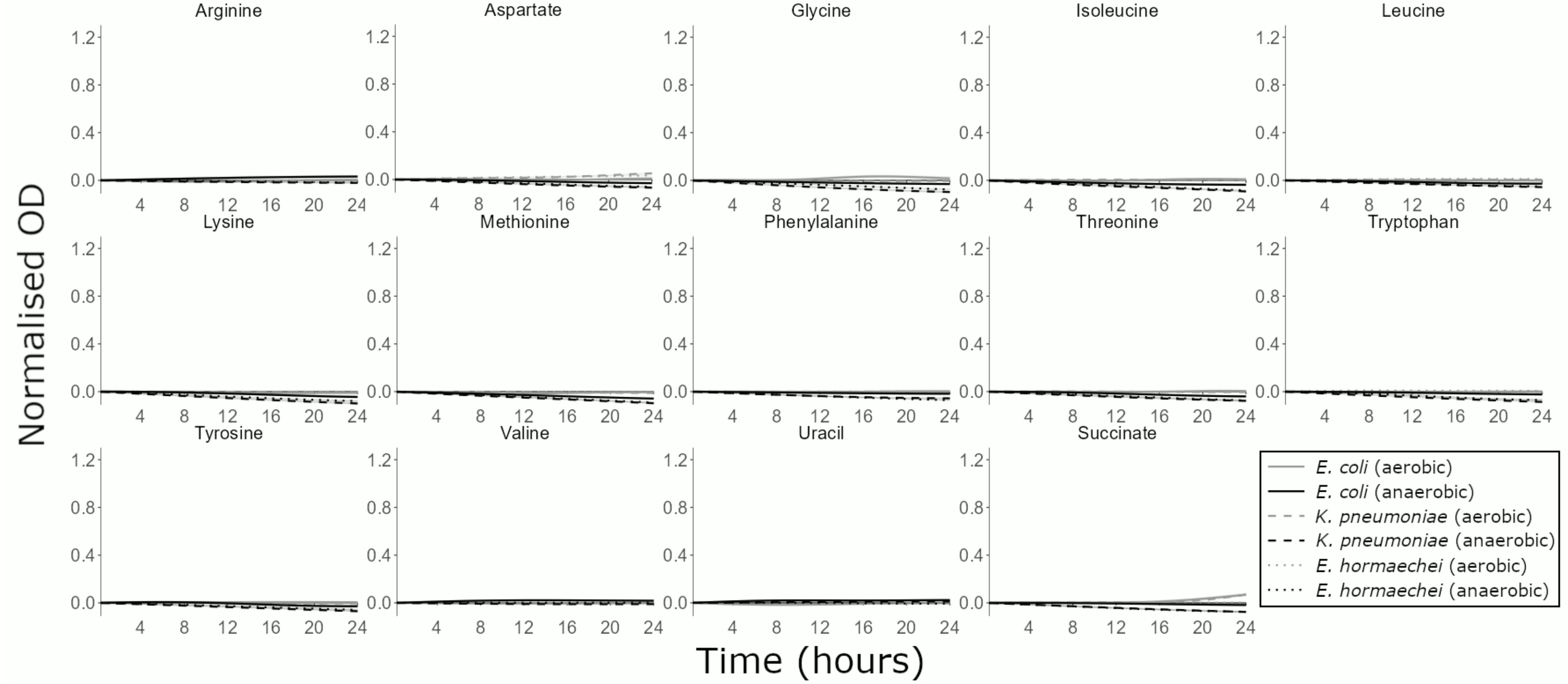

Figure S11: CRE growth was not supported by some individual carbon sources that were elevated in antibiotic-treated faecal microbiota. AMiGA-predicted growth curves for *E. coli* ST617, *K. pneumoniae* ST1026, and *E. hormaechei* ST278 grown on M9 minimal medium supplemented with a single carbon source (or water as the no carbon control) under anaerobic or aerobic conditions. Growth of each isolate was measured with 6 replicates in 2-3 independent experiments. The predicted mean of growth is shown with bold lines and the predicted 95% credible intervals are shown with the shaded bands. Optical density, OD. Source data are provided as a Source Data file.

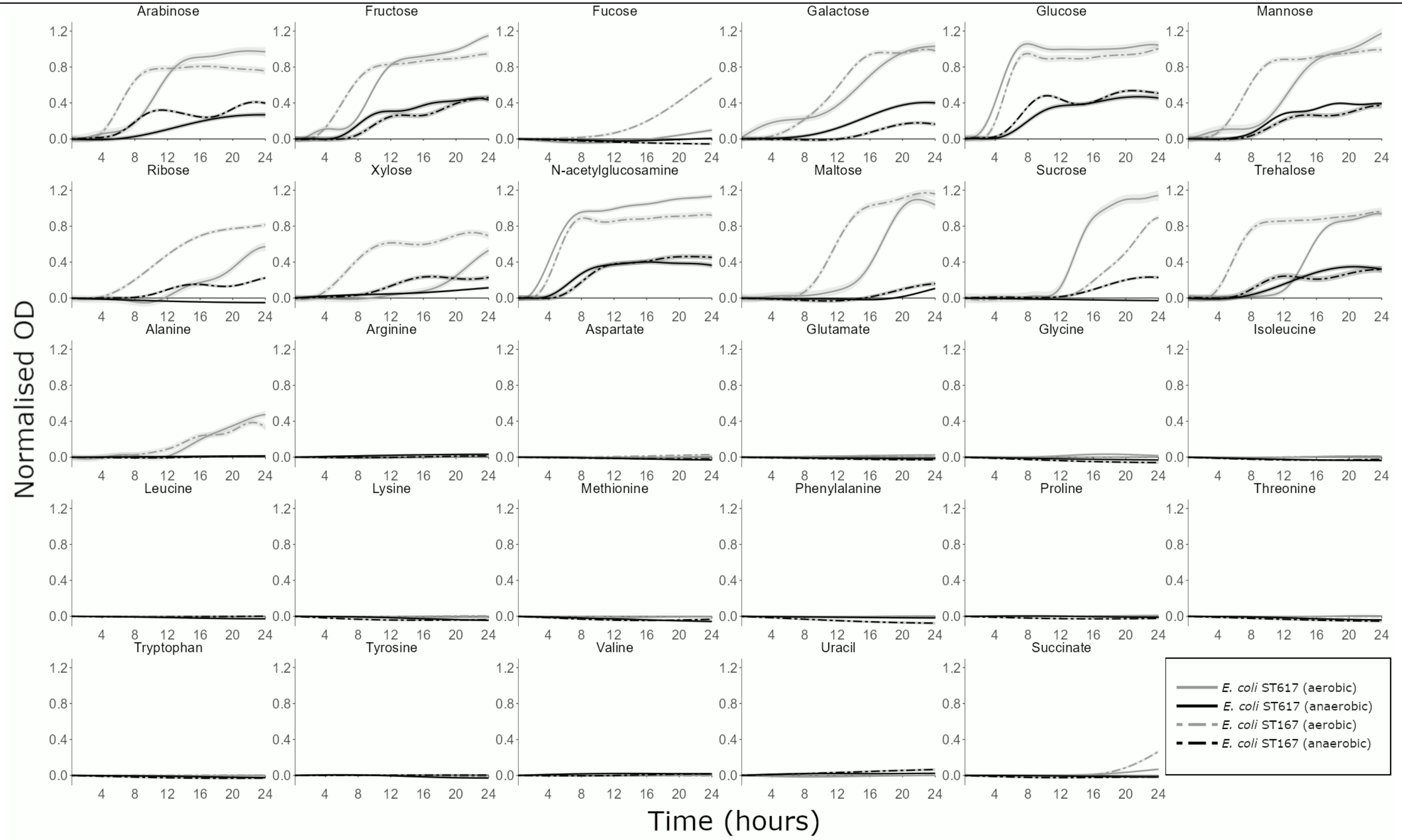

Figure S12: Carbapenem-resistant *E. coli* growth was supported by many individual carbon sources that were elevated in antibiotic-treated faecal microbiota but showed some differences between strains. AMiGA-predicted growth curves for *E. coli* ST617 and *E. coli* ST167 grown on M9 minimal medium supplemented with a single carbon source (or water as the no carbon control) under anaerobic or aerobic conditions. Growth of each isolate was measured with 6 replicates in 2-3 independent experiments. The predicted mean of growth is shown with bold lines and the predicted 95% credible intervals are shown with the shaded bands. Optical density, OD. Source data are provided as a Source Data file.

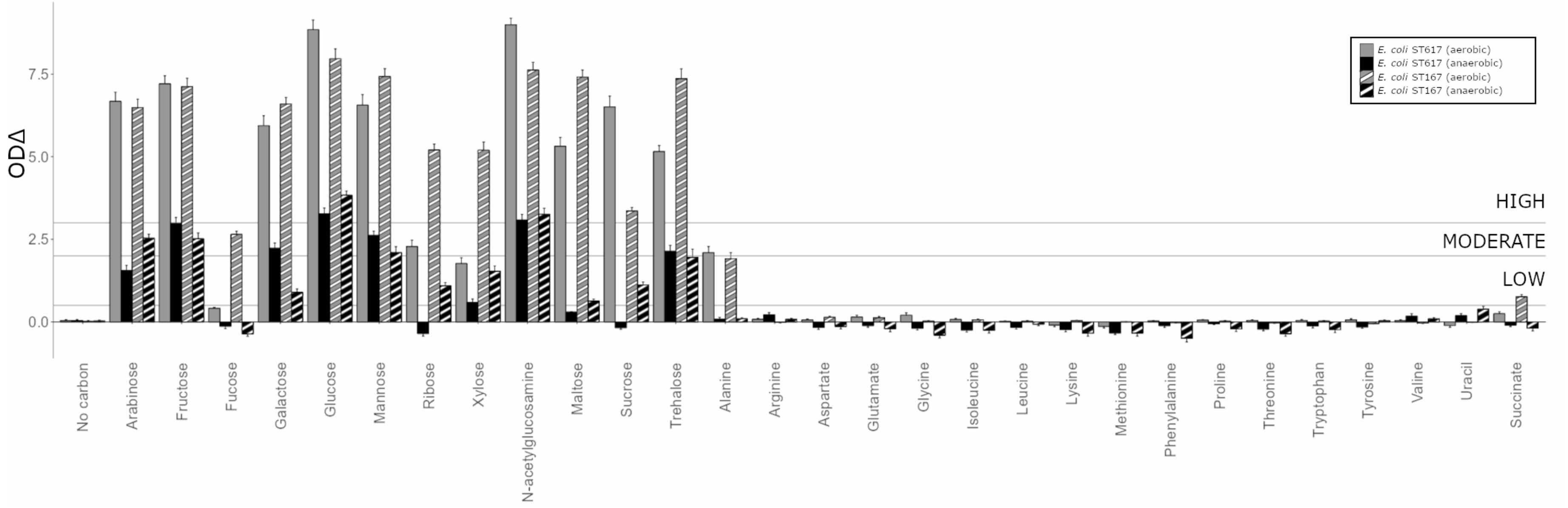

Figure S13: Individual carbon sources support the growth of carbapenem-resistant *E. coli* at high, moderate, or low levels. Functional differences (ODΔ) between growth on a carbon source versus growth on the no carbon control for *E. coli* ST617 and *E. coli* ST167 grown under anaerobic or aerobic conditions are summarised with the sum of functional differences quantifying the magnitude of differences between two growth curves. ODΔ >3 indicated high growth, ODΔ between 2-3 indicated moderate growth, ODΔ between 0.5-2 indicated low growth, ODΔ <0.5 indicated negligible or no growth. Error bars indicate 95% confidence intervals. Growth of each isolate was measured with 6 replicates in 2-3 independent experiments. Source data are provided as a Source Data file.

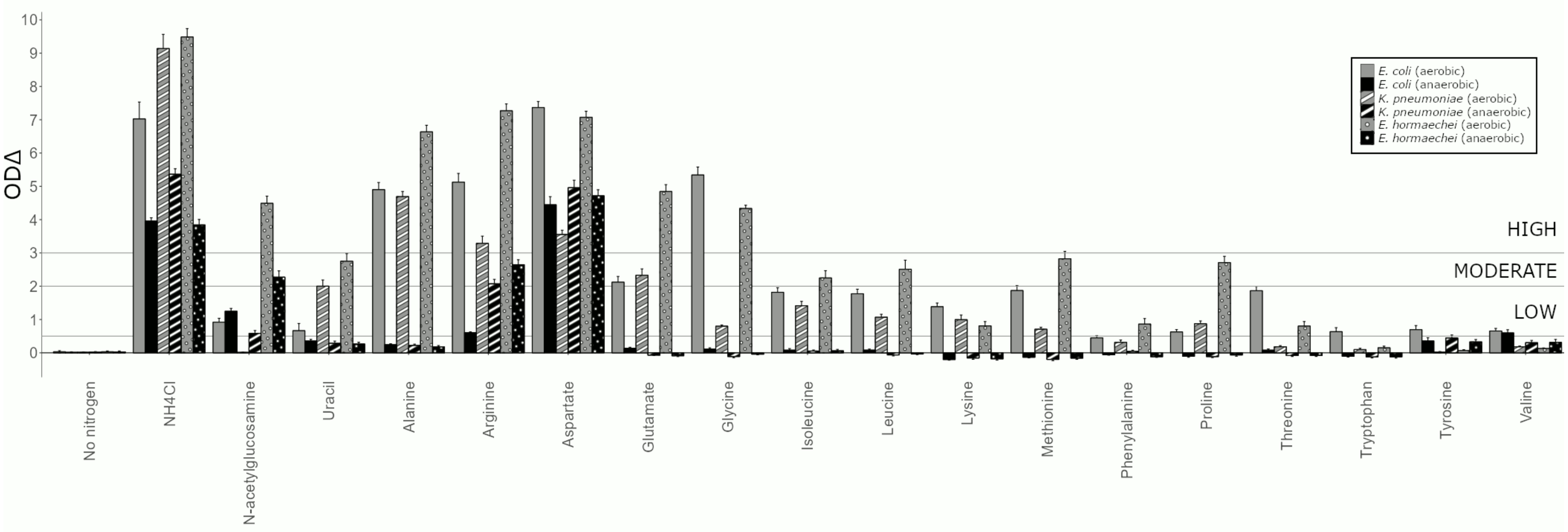

Figure S14: Individual nitrogen sources support the growth of CRE at high, moderate, or low levels. Functional differences (ODΔ) between growth on a nitrogen source versus growth on the no nitrogen control for *E. coli* ST617, *K. pneumoniae* ST1026, and *E. hormaechei* ST278 grown under anaerobic or aerobic conditions are summarised with the sum of functional differences quantifying the magnitude of differences between two growth curves. ODΔ >3 indicated high growth, ODΔ between 2-3 indicated moderate growth, ODΔ between 0.5-2 indicated low growth, ODΔ <0.5 indicated negligible or no growth. Error bars indicate 95% confidence intervals. Growth of each isolate was measured with 6 replicates in 2-3 independent experiments. Source data are provided as a Source Data file.

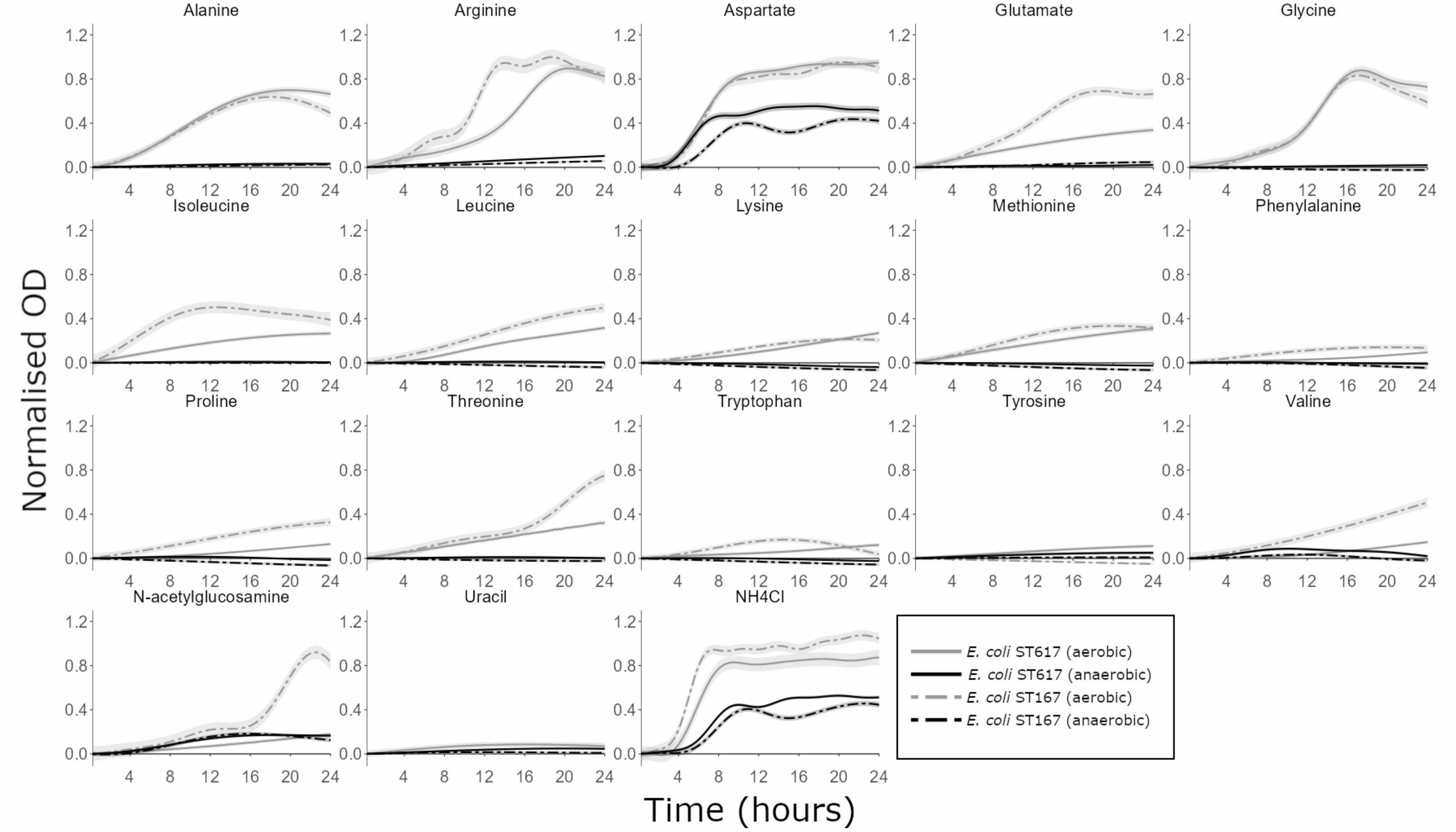

Figure S15: Carbapenem-resistant *E. coli* growth was supported by individual nitrogen sources that were elevated in antibiotic-treated faecal microbiota but showed some differences between strains. AMiGA-predicted growth curves for *E. coli* ST617 and *E. coli* ST167 grown on M9 minimal medium supplemented with a single nitrogen source (or water as the no nitrogen control) under anaerobic or aerobic conditions. Growth of each isolate was measured with 6 replicates in 2-3 independent experiments. The predicted mean of growth is shown with bold lines and the predicted 95% credible intervals are shown with the shaded bands. NH_4Cl was used as a positive control for growth as a sole nitrogen source. Optical density, OD. Source data are provided as a Source Data file.

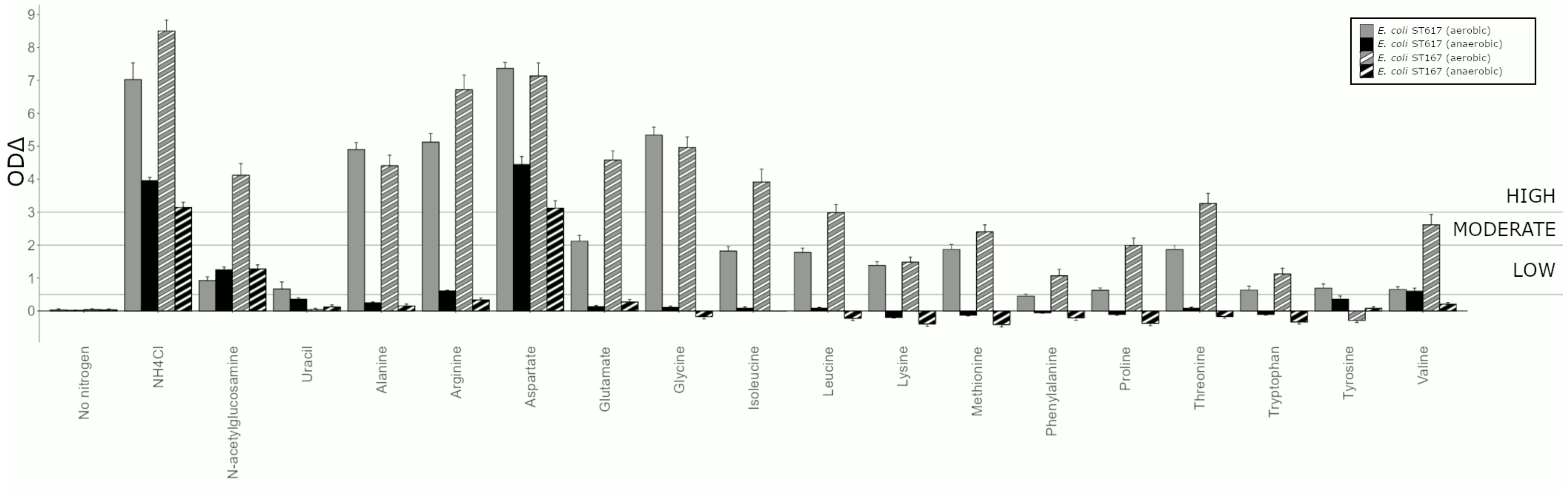

Figure S16: Individual nitrogen sources support the growth of carbapenem-resistant *E. coli* at high, moderate, or low levels. Functional differences (ODΔ) between growth on a nitrogen source versus growth on the no nitrogen control for *E. coli* ST617 and *E. coli* ST167 grown under anaerobic or aerobic conditions are summarised with the sum of functional differences quantifying the magnitude of differences between two growth curves. ODΔ >3 indicated high growth, ODΔ between 2-3 indicated moderate growth, ODΔ between 0.5-2 indicated low growth, ODΔ <0.5 indicated negligible or no growth. Error bars indicate 95% confidence intervals. Growth of each isolate was measured with 6 replicates in 2-3 independent experiments. Source data are provided as a Source Data file.

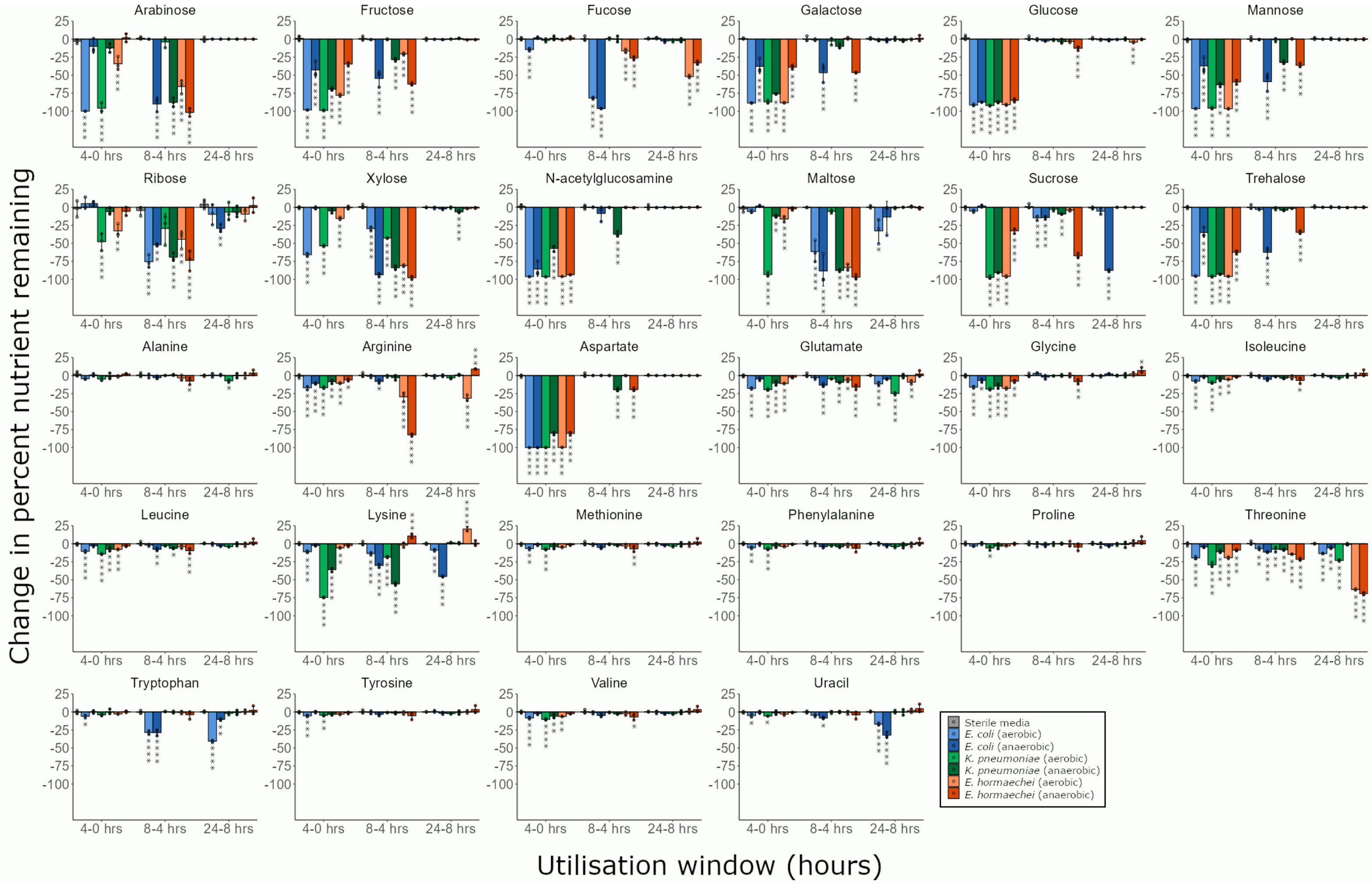

Figure S17: Order of nutrient utilisation varies by CRE isolate and by the presence or absence of oxygen. Percent change in nutrients between subsequent time points by *E. coli* ST617, *K. pneumoniae* ST1026, or *E. hormaechei* ST278 grown under anaerobic or aerobic conditions in M9 minimal medium containing a mixture of 0.015% of each nutrient. Nutrient concentration was measured by ^{1}H-NMR spectroscopy. Change in the percent of the nutrient remaining was calculated by subtracting the percent nutrient remaining at a time point from the percent nutrient remaining at the previous time point. Two-way mixed ANOVA followed by pairwise comparisons with Bonferroni correction. * = P ≤ 0.05, ** = P ≤ 0.01, *** = P ≤ 0.001, **** = P ≤ 0.0001, n=3 replicates for each CRE isolate, n=4 replicates for sterile media controls. Data are presented as mean values +/- SD. Source data and P values are provided in a Source Data file.

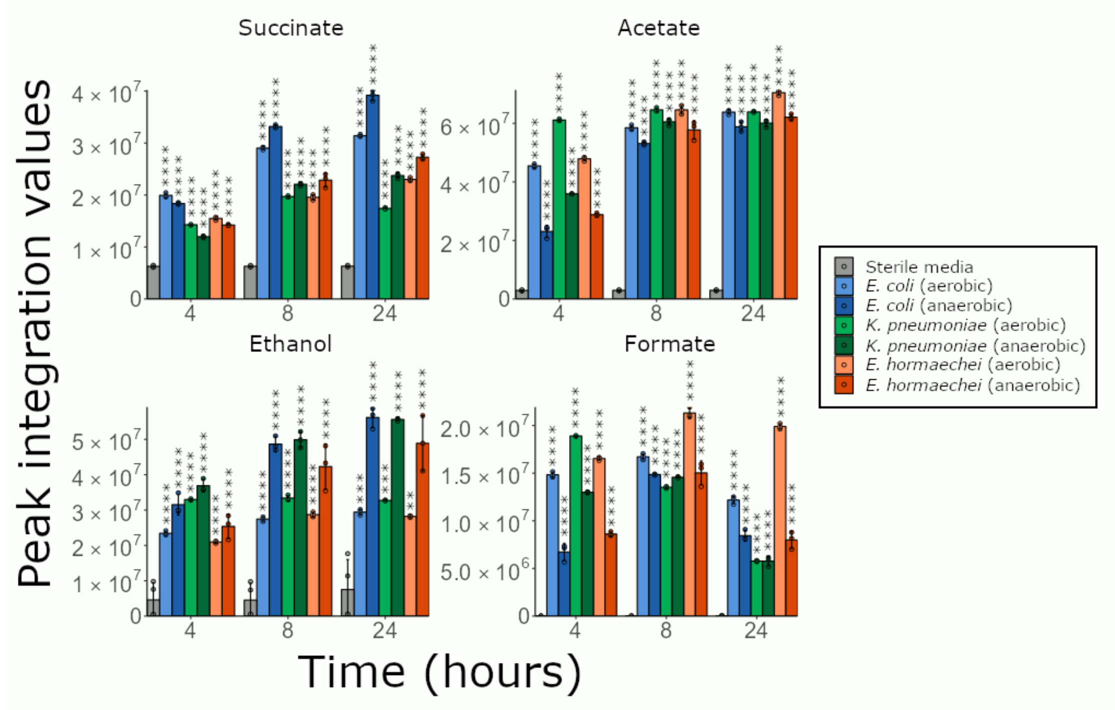

Figure S18: CRE isolates produce metabolites following incubation with a mixture of nutrients. Metabolite production by *E. coli* ST617, *K. pneumoniae* ST1026, or *E. hormaechei* ST278 grown under anaerobic or aerobic conditions in a minimal medium containing a mixture of 0.015% of each nutrient. Metabolite production was measured by ^{1}H-NMR spectroscopy. The metabolite concentration was measured by the integration of a representative peak in the NMR spectrum after incubation for 4hrs, 8 hrs, and 24 hrs. Two-way mixed ANOVA followed by pairwise comparisons with Bonferroni correction. * = P ≤ 0.05, ** = P ≤ 0.01, *** = P ≤ 0.001, **** = P ≤ 0.0001, n=3 replicates for each CRE isolate. Data are presented as mean values +/- SD. Source data and P values are provided in a Source Data file.

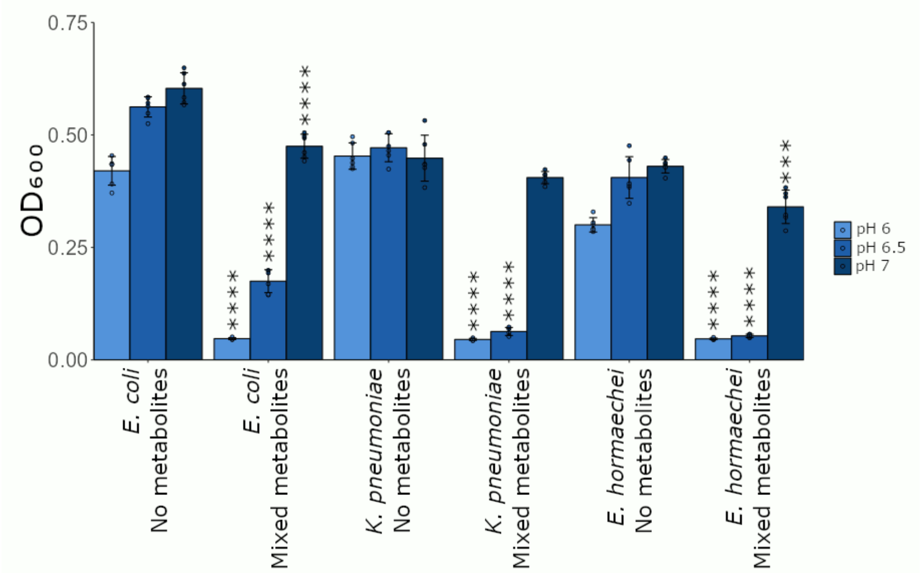

Figure S19: CRE growth was inhibited by a mixture of microbial metabolites (that were decreased in antibiotic-treated faecal microbiota) when grown in a minimal medium supplemented with nutrients (that were increased in antibiotic-treated faecal microbiota). *E. coli* ST617, *K. pneumoniae* ST1026, or *E. hormaechei* ST278 were grown in M9 minimal medium supplemented with a metabolite mixture (mimicking average human faecal concentrations) or unsupplemented (no metabolite control) at pH 6, 6.5, or 7. Growth of each isolate was measured with 6 replicates in 2 independent experiments. Unpaired t-test (two-sided) comparing metabolite mixture to the no metabolite control at the corresponding pH. *** = P ≤ 0.001, **** = P ≤ 0.0001. Data are presented as mean values +/- SD. Optical density at 600 nm, OD_{600}. Source data and P values are provided in a Source Data file.

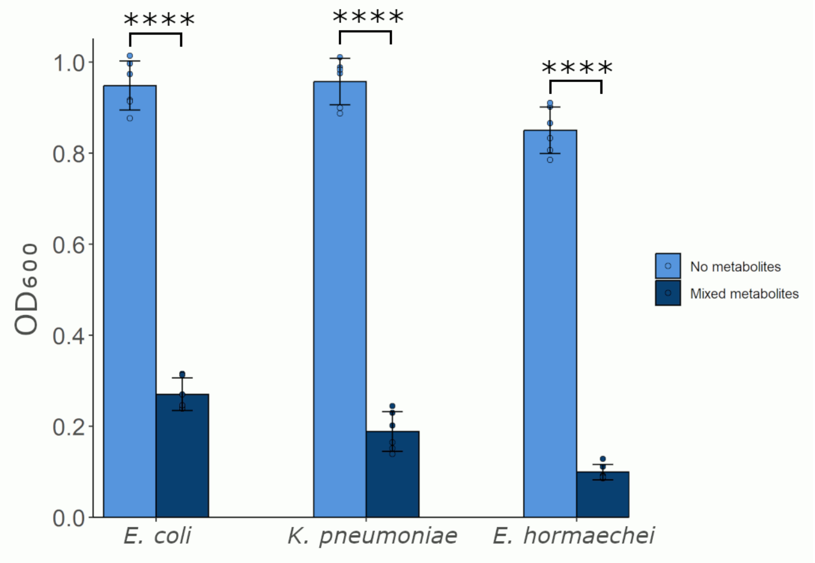

Figure S20: CRE growth was inhibited by a mixture of acetate, propionate, butyrate, and valerate. CRE growth was inhibited by a mixture of acetate, propionate, butyrate, and valerate at concentrations mimicking the average concentrations measured in human faeces. *E. coli* ST617, *K. pneumoniae* ST1026, or *E. hormaechei* ST278 were grown in LB supplemented with the metabolite mixture or unsupplemented (no metabolite control) at pH 6.5. Growth of each isolate was measured with 6 replicates in 2 independent experiments. Unpaired t-test (two-sided) comparing metabolite mixture to the no metabolite control. $P \leq 0.0001$ for *E. coli*, *K. pneumoniae*, and *E. hormaechei* (****). Data are presented as mean values +/- SD. Optical density at 600 nm, OD_{600}. Source data are provided as a Source Data file.

SUPPLEMENTARY TABLES

Table S1: Concentration of metabolites measured in human faeces from 12 healthy donors. Source data are provided as a Source Data file.

Metabolite	Lowest concentration (mM)	Average concentration (mM)	Highest concentration (mM)
Formate	0.07	0.11	0.17
Acetate	10.54	64.08	122.73
Propionate	5.68	16.10	34.69
Butyrate	2.94	16.38	38.75
Valerate	0.52	3.67	12.01
Isobutyrate	0.33	2.20	5.28
Isovalerate	0.48	2.00	4.55
Lactate	0.26	0.67	1.20
5-aminovalerate	0.29	1.06	4.15
Ethanol	0.64	10.46	59.62

Table S2: Concentration of metabolites tested in the metabolite inhibition assays, based on measurements from human faecal samples from 12 healthy donors (found in Table S1). Source data are provided as a Source Data file.

Metabolite	Lowest concentration (mM)	Average concentration (mM)	Highest concentration (mM)
Formate	0.05	0.10	0.15
Acetate	10	65	120
Propionate	5	15	35
Butyrate	3	15	40
Valerate	0.5	3.5	12
Isobutyrate	0.5	2	5
Isovalerate	0.5	2	5
Lactate	0.3	0.7	1.2
5-aminovalerate	0.3	1	4
Ethanol	0.6	10	60

Table S3: Reagents and oligonucleotides used in this study.

Reagent	Company	Code
3-Trimethylsilylpropionic-2,2,3,3-d_4 acid sodium salt	Merck	269913-1G
5-aminovalerate	Merck	123188-5G
Acetic Acid	Merck	A6283-100ML
Ammonium chloride	Fluorochem	044722-1KG
Arabinogalactan	TCI Europe	A1328-100G
Bile Salts	Fisher Scientific	10128872
Brilliance CRE agar	Fisher Scientific	13205349
Butyric Acid	Merck	B103500-100ML
Calcium Chloride Anhydrous	Fisher Scientific	10515671
Casein	VWR	A13707.36
Casein Hydrolysate	Merck	22090-100G
Deuterium Oxide	Goss Scientific	DLM-4-100G
D-Fructose	Merck	F0127-100G
D-Galactose	Fluorochem	218380-25G
D-Glucose	VWR	101174Y
D-Maltose	TCI Europe	M0037-25G
D-Mannose	TCI Europe	M0037-25G
DNeasy PowerLyzer PowerSoil Kit	Qiagen	12855-100
D-Ribose	Fluorochem	078886-25G
D-Trehalose dihydrate	Fluorochem	243212-25G
D-Xylose	Fluorochem	209047-100G
Escherichia coli DNA	Merck	D4889-1UN
Ethanol	Fisher Scientific	10437341
Formic Acid	VWR	20320.295
Hemin	Merck	51280-1G
Inulin	VWR	A18425.18
Iron (II) Sulfate Heptahydrate	Merck	215422-250G
Isobutyric Acid	Merck	I1754-100ML
Isovaleric Acid	Merck	129542-100ML
L-Alanine	TCI Europe	A0179-25G

L-Arabinose	Fluorochem	078969-25G
L-Arginine hydrochloride	TCI Europe	A0528-25G
L-Aspartic acid	TCI Europe	A0546-25G
LB Broth	Merck	L3022-1KG
L-Cysteine Hydrochloride Monohydrate	Merck	C7880-100G
L-Fucose	Fluorochem	216288-5G
L-Glutamic acid	TCI Europe	G0059-25G
L-Glycine	TCI Europe	G0099-25G
L-Isoleucine	TCI Europe	I0181-25G
L-Leucine	TCI Europe	L0029-25G
L-Lysine hydrochloride	TCI Europe	L0071-25G
L-Methionine	TCI Europe	M0099-25G
L-Phenylalanine	TCI Europe	P0134-25G
L-Proline	TCI Europe	P0481-25G
L-Threonine	TCI Europe	T0230-25G
L-Tryptophan	TCI Europe	T0541-25G
L-Tyrosine	TCI Europe	T0550-25G
L-Valine	TCI Europe	V0014-25G
Magnesium Sulphate Heptahydrate	Fisher Scientific	10424511
Menadione	Merck	M9429-25G
Mucin from porcine stomach Type II	Merck	M2378-500G
N-acetylglucosamine	TCI Europe	A0092-25G
PCR Grade Water	Merck	3315932001
Pectin Citrus	VWR	J61021.36
Peptone Water	Merck	70179-500G
Platinum Supermix with ROX	Life Technologies	11730017
Potassium Phosphate Dibasic	Merck	P3786-500G
Potassium Phosphate Monobasic	Merck	P5655-500G
Propionic Acid	Merck	P1386-500ML
Proteose Peptone	Merck	82450-100G
Sodium Azide	Merck	71289-5G
Sodium Bicarbonate	Merck	S6014-500G

Sodium Chloride	Merck	S7653-1KG
Sodium DL-lactate	Merck	71720-5G
Sodium Phosphate Dibasic	Merck	71640-250G
Sodium Phosphate Monobasic	Fisher Scientific	389872500
Sodium Pyruvate	SLS	P2256-5G
Sodium Succinate Dibasic Hexahydrate	SLS	S2378-100G
Soluble Starch	SLS	CHE3622
Starch	Merck	S5127-5KG
Sucrose	Merck	84100-250G
Triacetin (aka glycerol triacetate)	Merck	525073-1L
Tributyrin (aka glycerol tributyrate)	Merck	W222305
Tripropionin (aka glycerol tripropionate)	Merck	W328618-1KG-K
Trivalerin (aka glycerol trivalerate)	Merck	93498-1G
Uracil	TCI Europe	U0013-25G
Valeric acid	Merck	240370-100ML
Xylan	Universal Biologicals	38500.02
Yeast Extract	Merck	70161-500G
16S sequencing primer: 28F-YM (forward primer, MiSeq adapter sequences in bold)	Eurofins	5'-**TCGTCGGCAGCGTCAGATGTGTATAAGAGACAG**GAGTTTGATYMTGGCTCAG-3'
16S sequencing primer: 28F-Borrellia (forward primer, MiSeq adapter sequences in bold)	Eurofins	5'-**TCGTCGGCAGCGTCAGATGTGTATAAGAGACAG**GAGTTTGATCCTGGCTTAG-3'
16S sequencing primer: 28F-Chloroflex (forward primer, MiSeq adapter sequences in bold)	Eurofins	5'-**TCGTCGGCAGCGTCAGATGTGTATAAGAGACAG**GAATTTGATCTTGGTTCAG-3'
16S sequencing primer: 28F-Bifdo (forward primer, MiSeq adapter sequences in bold)	Eurofins	5'-**TCGTCGGCAGCGTCAGATGTGTATAAGAGACAG**GGGTTCGATTCTGGCTCAG-3'
16S sequencing primer: 388R (reverse primer, MiSeq adapter sequences in bold)	Eurofins	5'-**GTCTCGTGGGCTCGGAGATGTGTATAAGAGACAG**TGCTGCCTCCCGTAGGAGT-3'
16S rRNA gene qPCR: BactQUANT forward primer	Eurofins	5′-CCTACGGGAGGCAGCA-3′
16S rRNA gene qPCR: BactQUANT reverse primer	Eurofins	5′-GGACTACCGGGTATCTAATC-3′
16S rRNA gene qPCR: BactQUANT probe	Life Technologies	4316034, [6FAM] 5′-CAGCAGCCGCGGTA-3′ [MGBNFQ]

Table S4: MICs measured for CRE strains used in this study. Source data are provided as a Source Data file.

Strain	Meropenem, mg/L	Imipenem, mg/L	Ertapenem, mg/L	Piperacillin/ tazobactam, mg/L
E. coli ST617	64 (resistant)	16 (resistant)	128 (resistant)	>128 (resistant)
K. pneumoniae ST1026	8 (intermediate)	4 (intermediate)	16-32 (resistant)	>128 (resistant)
E. hormaechei ST278	16 (resistant)	4-8 (intermediate/resistant)	32 (resistant)	>128 (resistant)
E. coli ST167	64 (resistant)	16-32 (resistant)	64-128 (resistant)	>128 (resistant)
E. coli ST410	2 (sensitive)	2-4 (sensitive/intermediate)	8 (resistant)	>128 (resistant)
K. pneumoniae ST258	>128 (resistant)	128 (resistant)	>128 (resistant)	>128 (resistant)
K. pneumoniae ST11	128 (resistant)	128 (resistant)	>128 (resistant)	>128 (resistant)

Table S5: Results from multivariate general linear model analysing the effects of faecal donor age and sex on the ^{1}H-NMR spectroscopy data from the antibiotic-naïve faecal culture experiments. ANOVA (two-sided) with Bonferroni correction for multiple comparisons. Source data are provided as a Source Data file.

Metabolite	Sex			Age		
	F	P value	Adjusted P value	F	P value	Adjusted P value
Formate	0.004	0.952	1.000	0.101	0.759	1.000
Acetate	0.013	0.911	1.000	0.024	0.881	1.000
Propionate	1.489	0.257	1.000	0.768	0.407	1.000
Butyrate	0.15	0.708	1.000	1.388	0.273	1.000
Valerate	0.278	0.612	1.000	0.045	0.837	1.000
Isobutyrate	1.737	0.224	1.000	0.125	0.733	1.000
Isovalerate	0.834	0.388	1.000	0.017	0.899	1.000
Lactate	0.015	0.906	1.000	0.214	0.656	1.000
5-aminovalerate	0.789	0.4	1.000	0.796	0.398	1.000
Ethanol	0.09	0.772	1.000	0.235	0.641	1.000

Table S6: Results from multivariate general linear model analysing the effects of faecal donor age and sex on the 16S rRNA gene sequencing data from the antibiotic-naïve faecal culture experiments. ANOVA (two-sided) with Bonferroni correction for multiple comparisons. Source data are provided as a Source Data file.

Family	Sex			Age		
	F	P value	Adjusted P value	F	P value	Adjusted P value
Bifidobacteriaceae	2.142	0.181	1.000	1.832	0.213	1.000
Atopobiaceae	0.597	0.462	1.000	0.064	0.807	1.000
Coriobacteriaceae	1.521	0.252	1.000	0.688	0.431	1.000
Coriobacteriales Incertae Sedis	1.565	0.246	1.000	0.292	0.603	1.000
Eggerthellaceae	0.563	0.474	1.000	1.385	0.273	1.000
Bacteroidaceae	1.18	0.309	1.000	1.307	0.286	1.000
Barnesiellaceae	0.024	0.880	1.000	0.178	0.684	1.000
Marinifilaceae	0.696	0.428	1.000	0.69	0.43	1.000
Muribaculaceae	1.778	0.219	1.000	29.045	0.001	0.035
Prevotellaceae	0.124	0.734	1.000	2.237	0.173	1.000
Rikenellaceae	0.095	0.766	1.000	0.018	0.896	1.000
Tannerellaceae	0.696	0.428	1.000	0.661	0.44	1.000
Desulfovibrionaceae	0	0.985	1.000	0.267	0.62	1.000
Erysipelatoclostridiaceae	0.157	0.702	1.000	0.056	0.818	1.000
Erysipelotrichaceae	1.527	0.252	1.000	0.697	0.428	1.000
Lactobacillaceae	1.563	0.247	1.000	0.63	0.45	1.000
Streptococcaceae	0.826	0.390	1.000	1.068	0.332	1.000
Christensenellaceae	0.49	0.504	1.000	0.281	0.61	1.000
Clostridiaceae	0.161	0.699	1.000	0.25	0.631	1.000
Lachnospiraceae	0.141	0.717	1.000	0.444	0.524	1.000
Monoglobaceae	0.536	0.485	1.000	1.455	0.262	1.000
Eubacterium coprostanoligenes group	0.519	0.492	1.000	0.098	0.763	1.000
Butyricicoccaceae	0.42	0.535	1.000	0.061	0.812	1.000
Oscillospiraceae	0.054	0.823	1.000	0.186	0.678	1.000
Ruminococcaceae	0.018	0.896	1.000	0.704	0.426	1.000
UCG 010	0.832	0.388	1.000	0.149	0.71	1.000
Peptostreptococcaceae	3.135	0.115	1.000	0.12	0.737	1.000
Acidaminococcaceae	0.146	0.713	1.000	5.698	0.044	1.000
Selenomonadaceae	1.642	0.236	1.000	0.025	0.878	1.000
Veillonellaceae	0.003	0.960	1.000	0.199	0.667	1.000
Sutterellaceae	0.177	0.685	1.000	0.659	0.44	1.000
Enterobacteriaceae	1.928	0.202	1.000	2.249	0.172	1.000
Akkermansiaceae	1.193	0.307	1.000	0.892	0.373	1.000
Unclassified	0.925	0.364	1.000	0.024	0.881	1.000
ASV other	0.271	0.617	1.000	0.147	0.711	1.000

Table S7: Results from multivariate general linear model analysing the effects of faecal donor age and sex on the ^{1}H-NMR spectroscopy data from human faeces (from the data summarised in Table S1). ANOVA (two-sided) with Bonferroni correction for multiple comparisons. Source data are provided as a Source Data file.

Metabolite	Sex			Age		
	F	P value	Adjusted P value	F	P value	Adjusted P value
Formate	1.584	0.240	1.000	0.921	0.362	1.000
Acetate	0.074	0.791	1.000	1.097	0.322	1.000
Propionate	1.409	0.266	1.000	0.144	0.713	1.000
Butyrate	0.299	0.598	1.000	0.118	0.739	1.000
Valerate	0.77	0.403	1.000	0.068	0.800	1.000
Isobutyrate	0.215	0.654	1.000	0.826	0.387	1.000
Isovalerate	0.016	0.902	1.000	0.885	0.371	1.000
Lactate	0.534	0.483	1.000	0.468	0.511	1.000
5-aminovalerate	0.462	0.514	1.000	0.314	0.589	1.000
Ethanol	0.423	0.532	1.000	2.137	0.178	1.000

SUPPLEMENTARY NOTE 1

Impact of faecal donor age and sex on ^{1}H-NMR spectroscopy and 16S rRNA gene sequencing data

Multivariate general linear models were used to analyse the ^{1}H-NMR spectroscopy data and 16S rRNA gene sequencing data from the antibiotic-naïve faecal cultures to determine whether age or sex of the donors impacted these measurements. We found no significant differences in any of the measured metabolites due to age or sex (Table S5). We found no significant differences in any of the bacterial families due to sex, however we found one bacterial family (*Muribaculaceae*) was affected by age (Table S6). However, *Muribaculaceae* was only detected in 1 of the 11 donors, and it was present in this donor at a very low level approaching the limit of detection. Moreover, we did not find a significant difference in *Muribaculaceae* abundance in any of the antibiotic-treated groups (Fig. 1a). Therefore, the potential influence of age on *Muribaculaceae* abundance did not impact the outcomes of this study.

A multivariate general linear model was also used to analyse the ^{1}H-NMR spectroscopy data from the donor faecal samples (data presented in Table S1) to determine whether age or sex of the donors impacted these measurements. There were no significant differences in any of the measured metabolites due to age or sex (Table S7).
