# Supplementary material for: Antibiotics promote intestinal growth of carbapenem-resistant Enterobacteriaceae by enriching nutrients and depleting microbial metabolites
Source: Nat Commun. 2023 Aug 22;14:5094. doi: 10.1038/s41467-023-40872-z (PMC10444851; doi:10.1038/s41467-023-40872-z)
Supplement: Supplementary file 3 — Description of Additional Supplementary Files [file 41467_2023_40872_MOESM3_ESM.pdf]

Description of Additional Supplementary Files:

Supplementary Data 1: Whole genome sequencing results and antimicrobial resistance genes detected from draft genomes of CRE patient isolates.
