# Supplementary material for: Antibiotics promote intestinal growth of carbapenem-resistant Enterobacteriaceae by enriching nutrients and depleting microbial metabolites
Source: Nat Commun. 2023 Aug 22;14:5094. doi: 10.1038/s41467-023-40872-z (PMC10444851; doi:10.1038/s41467-023-40872-z)
Supplement: Supplementary file 5 — Reporting Summary [file 41467_2023_40872_MOESM5_ESM.pdf]

nature portfolio

Corresponding author(s): Dr Julie A. K. McDonald

Last updated by author(s): Aug 5, 2023

Reporting Summary

Nature Portfolio wishes to improve the reproducibility of the work that we publish. This form provides structure for consistency and transparency in reporting. For further information on Nature Portfolio policies, see our Editorial Policies and the Editorial Policy Checklist.

Statistics

For all statistical analyses, confirm that the following items are present in the figure legend, table legend, main text, or Methods section.

n/a	Confirmed	
☐	☒	The exact sample size (*n*) for each experimental group/condition, given as a discrete number and unit of measurement
☐	☒	A statement on whether measurements were taken from distinct samples or whether the same sample was measured repeatedly
☐	☒	The statistical test(s) used AND whether they are one- or two-sided<br>*Only common tests should be described solely by name; describe more complex techniques in the Methods section.*
☐	☒	A description of all covariates tested
☐	☒	A description of any assumptions or corrections, such as tests of normality and adjustment for multiple comparisons
☐	☒	A full description of the statistical parameters including central tendency (e.g. means) or other basic estimates (e.g. regression coefficient) AND variation (e.g. standard deviation) or associated estimates of uncertainty (e.g. confidence intervals)
☐	☒	For null hypothesis testing, the test statistic (e.g. *F*, *t*, *r*) with confidence intervals, effect sizes, degrees of freedom and *P* value noted<br>*Give P values as exact values whenever suitable.*
☒	☐	For Bayesian analysis, information on the choice of priors and Markov chain Monte Carlo settings
☒	☐	For hierarchical and complex designs, identification of the appropriate level for tests and full reporting of outcomes
☒	☐	Estimates of effect sizes (e.g. Cohen's *d*, Pearson's *r*), indicating how they were calculated

Our web collection on statistics for biologists contains articles on many of the points above.

Software and code

Policy information about availability of computer code

Data collection	16S rRNA gene sequencing data was collected using the MiSeq Control Software. 16S rRNA gene qPCR data was collected using the Applied Biosystems StepOnePlus Software, NMR spectra were collected using Bruker's IconNMR component within TopSpin.
Data analysis	Statistical tests were performed using IBM SPSS Statistics Software 27 (IBM Corp, Armonk, New York), GraphPad Prism 9.4.1 (La Jolla, California), or in R (v4.2.2).<br><br>16S rRNA gene sequencing data were imported into R and processed using the standard DADA2 pipeline (version 1.18.0). 16S rRNA gene sequencing data (weighted as absolute abundances) were analysed using Wilcoxon signed rank test with Benjamini & Hochberg FDR correction using the DA.wil function within in the DAtest R package version 2.7.18.<br><br>NMR spectra were processed in Topspin (v3.2.6), imported into MATLAB r2019b, and processed using a custom script available in the Figshare repository at https://doi.org/10.6084/m9.figshare.21948233 using the code available at the Zenodo repository at https://doi.org/10.5281/zenodo.3077413. Peak integration values from the 1H-NMR data were analysed using Wilcoxon signed rank test with Benjamini & Hochberg FDR correction using the DA.wil function within in the DAtest R package version 2.7.18 or a paired t-test (two-sided) with Benjamini & Hochberg FDR correction using the pairwise_t_test function within the rstatix R package version 0.7.1. Metabolite concentrations were also measured from NMR spectra using the Chenomx NMR Suite (v8.6).<br><br>Multivariate general linear models were analysed in IBM SPSS Statistics Software 27 to determine whether faecal donor age or sex impacted the 1H-NMR spectroscopy or 16S rRNA gene sequencing measurements, using the 1H-NMR metabolite measurements or 16S rRNA gene sequencing read counts as the dependent variables, sex as a fixed factor, and age as a covariate.

Regularised canonical correlation analysis (rCCA) was used to correlate the 16S rRNA gene sequencing data and 1H-NMR data using the mixOmics R package version 6.22.0.

Partial Spearman correlations were calculated between the 1H-NMR spectroscopy data and 16S rRNA gene sequencing data from the faecal culture experiments using IBM SPSS Statistics Software 27 and plotted using the corrplot function within the corrplot R package version 0.92.

For the carbon and nitrogen utilisation assays the growth curves were analysed in Python (v3.6.5) using the AMiGA software (available at https://github.com/firasmidani/amiga, PMID: 34254821).

For the whole genome sequencing data from the CRE patient isolates, raw reads were trimmed to remove sequences and low-quality bases with Trimmomatic v0.39. Bacterial species from assembled genomes was confirmed using Kraken2 v2.0.8-beta and its full bacterial database. Draft genomes were generated de novo using SPAdes v3.15.2. Assembly statistics were checked using QUAST v5.0.2. Acquired antimicrobial resistance genes were detected from draft genomes using ABRicate v1.0.1 with the ResFinder database. Multi locus sequence types (MLST) were determined from draft genomes using MLST v2.19.0. The following MLST databases were used to determine MLST: E. hormaechei – Enterobacter cloacae MLST scheme, E. coli – Achtman's E. coli MLST, K. pneumoniae – Klebsiella pneumoniae MLST scheme.

For manuscripts utilizing custom algorithms or software that are central to the research but not yet described in published literature, software must be made available to editors and reviewers. We strongly encourage code deposition in a community repository (e.g. GitHub). See the Nature Portfolio guidelines for submitting code & software for further information.

Data

Policy information about availability of data

All manuscripts must include a data availability statement. This statement should provide the following information, where applicable:

- Accession codes, unique identifiers, or web links for publicly available datasets
- A description of any restrictions on data availability
- For clinical datasets or third party data, please ensure that the statement adheres to our policy

Source data are provided with this paper. 16S rRNA gene sequencing, 16S rRNA gene qPCR, and 1H-NMR datasets generated in this study have been deposited into the Figshare repository at https://doi.org/10.6084/m9.figshare.21948233. Raw reads for whole genome sequences for the CRE patient isolates have been deposited to the European Nucleotide Archive (ENA) under the BioProject PRJEB60914 (https://www.ebi.ac.uk/ena/browser/view/PRJEB60914) with isolate accession numbers NDM-5 E. coli ST617 (ERR11452083, https://www.ebi.ac.uk/ena/browser/view/ERR11452083), NDM-1 K. pneumoniae ST1026 (ERR11452082, https://www.ebi.ac.uk/ena/browser/view/ERR11452082), and NDM-1 E. hormaechei ST278 (ERR11452081, https://www.ebi.ac.uk/ena/browser/view/ERR11452081) and strains are available upon request. Raw reads for the 16S rRNA gene sequencing data have also been deposited to the ENA under the BioProject PRJEB60914 (https://www.ebi.ac.uk/ena/browser/view/PRJEB60914) with sample accession numbers and links found in the Figure 1 Source Data file.

For the whole genome sequencing data from the CRE patient isolates, the Kraken bacterial database is available at http://ccb.jhu.edu/software/kraken2/, the ResFinder database is available at https://cge.food.dtu.dk/services/ResFinder/, and the MLST databases are available at https://pubmlst.org and https://bigsdb.pasteur.fr/klebsiella/. For the 16S rRNA gene sequencing data the SILVA bacterial database version 138.1 is available at https://www.arb-silva.de/. For the 1H-NMR spectroscopy data the compound database in the Chenomx NMR Suite v9.02 is available at https://www.chenomx.com/.

Human research participants

Policy information about studies involving human research participants and Sex and Gender in Research.

Reporting on sex and gender	Male and female participants were used equally for the study as there is no biological reason to favour one sex over the other. Findings apply to both sexes and both sexes were included in this study. Sex was determined based on self-reporting. In the first faecal culture experiment, 6 males and 5 females provided faecal donations to seed the faecal cultures. In the second faecal culture experiment, 2 males and 3 females provided faecal donations to seed the faecal cultures. For the donors that provided faecal samples to measure the concentration of specific microbial metabolites, 5 males and 7 females provided faecal samples.
Population characteristics	Donors were between 18-65 years old and had not received antibiotic treatment in the 6+ months prior to donation.
Recruitment	Healthy donors were approached and asked if they would be interested in participating in this study. Informed consent was obtained from participants with information provided in the form of an Information Sheet detailing the research objectives and the samples needed. Samples were collected after the participant had agreed to participate in the study, had read the Information Sheet, and had signed the Informed Consent form.<br><br>As healthy donors were approached and asked to participate in our study (rather than through open advertisement to recruit donors), this study was not affected by self-selection bias. There is nothing in the donor recruitment process that would favour a particular sex, ethnicity, or other characteristic over others and participation was entirely voluntary.
Ethics oversight	Ethical approval was received by the London - Queen Square Research Ethics Committee (19/LO/0112) and the South Central - Oxford C Research Ethics Committee (16/SC/0021 and 20/SC/0389).

Note that full information on the approval of the study protocol must also be provided in the manuscript.

Field-specific reporting

Please select the one below that is the best fit for your research. If you are not sure, read the appropriate sections before making your selection.

- [x] Life sciences
- [ ] Behavioural & social sciences
- [ ] Ecological, evolutionary & environmental sciences

For a reference copy of the document with all sections, see nature.com/documents/nr-reporting-summary-flat.pdf

Life sciences study design

All studies must disclose on these points even when the disclosure is negative.

Sample size	Power calculations were used to determine sample size using the appropriate statistical test (e.g. paired or unpaired t-test), an alpha of 0.05, a power of 0.80, and an effect size calculated from an appropriate existing data set.
Data exclusions	For the mouse dataset, one NMR spectrum (from day 2) had more than 50% of integrated peaks identified as an outlier or extreme outlier (using the identify_outliers function within the rstatix library within R) and was excluded. As paired t-tests were conducted, samples from this mouse were also excluded at the other time points (as paired t-test can only use cases that have non-missing values).<br><br>One sample was excluded from the TZP-treated faecal culture for E. coli ST410 outlined in Figure 1c as this was an extreme significant outlier (as calculated using the Grubbs' test in GraphPad).
Replication	Experiments were repeated the number of times indicated in the figure legends. All attempts at replication were successful.
Randomization	Mice were randomly allocated into different groups.<br><br>For the human faecal culture experiments, each donor faecal sample was homogenised and split to seed the antibiotic-naïve group and each antibiotic-treated group. This means that each donor was allocated into all of the treatment groups.<br><br>For the human faecal samples used to determine a minimum, average, and maximum concentration of each metabolite, there were no treatment groups.
Blinding	Investigators were not blinded for in vitro assays or 1H-NMR preparation because the same investigator was doing group allocation during data collection. Blinding was not used for animal experiments because the investigators needed to know the treatment groups in order to administer the interventions. 16S rRNA gene sequencing library preparation was blinded.

Reporting for specific materials, systems and methods

We require information from authors about some types of materials, experimental systems and methods used in many studies. Here, indicate whether each material, system or method listed is relevant to your study. If you are not sure if a list item applies to your research, read the appropriate section before selecting a response.

Materials & experimental systems

n/a	Involved in the study	
x		Antibodies
x		Eukaryotic cell lines
x		Palaeontology and archaeology
	x	Animals and other organisms
x		Clinical data
x		Dual use research of concern

Methods

n/a	Involved in the study	
x		ChIP-seq
x		Flow cytometry
x		MRI-based neuroimaging

Animals and other research organisms

Policy information about studies involving animals; ARRIVE guidelines recommended for reporting animal research, and Sex and Gender in Research

Laboratory animals	Eight- to ten-week-old female wild-type C57BL/6 mice were purchased from Envigo (Huntingdon, UK) and acclimatised for 1 week prior to the start of the experiment. Each test group contained 5 mice that were housed 5 per cage (in individually ventilated cages). Mice were provided autoclaved food (RM1, Special Diet Services, Essex, UK), water (provided ad libitum), and bedding (Aspen chip 2 bedding, NEPCO, Warrensburg, New York). Mice were maintained at 20–22°C and 45-65% humidity in a 12-hour light and 12-hour dark cycle (all interventions were performed during the light cycle).
Wild animals	The study did not involve wild animals.

Reporting on sex	Female C57BL/6 mice were used in this study. The findings of this study apply to both sexes.
Field-collected samples	The study did not involve samples collected from the field.
Ethics oversight	Ethical approval for mouse experiments was received from the Imperial College London Animal Welfare and Ethical Review Body (PF93C158E).

Note that full information on the approval of the study protocol must also be provided in the manuscript.
